# Multiple ion channel block by the cation channel inhibitor SKF‐96365 in myocytes from the rabbit atrioventricular node

**DOI:** 10.14814/phy2.12819

**Published:** 2016-06-10

**Authors:** Hongwei Cheng, Alexander E. Curtis, Claire Fellingham, Jules C. Hancox

**Affiliations:** ^1^ Cardiovascular Research LaboratoriesSchool of Physiology, Pharmacology and NeuroscienceUniversity of BristolBristolUK

**Keywords:** Atrioventricular node, AV node, AVN, background current, calcium current, hyperpolarization‐activated current, *I*_B,Na_, *I*_Ca,L_, *I*_f_, *I*_Kr_, pacemaking, rapid delayed rectifier, SKF‐96365

## Abstract

The atrioventricular node (AVN) of the cardiac conduction system coordinates atrial–ventricular excitation and can act as a subsidiary pacemaker. Recent evidence suggests that an inward background sodium current, *I*_B,Na_, carried by nonselective cation channels (NSCCs), contributes to AVN cell pacemaking. The study of the physiological contribution of *I*_B,Na_ has been hampered, however, by a lack of selective pharmacological antagonists. This study investigated effects of the NSCC inhibitor SKF‐96365 on spontaneous activity, *I*_B,Na_, and other ionic currents in AVN cells isolated from the rabbit. Whole‐cell patch‐clamp recordings of action potentials (APs) and ionic currents were made at 35–37°C. A concentration of 10 *μ*mol/L SKF‐96365 slowed spontaneous action potential rate by 13.9 ± 5.3% (*n* = 8) and slope of the diastolic depolarization from 158.1 ± 30.5 to 86.8 ± 30.5 mV sec^−1^ (*P *< 0.01; *n* = 8). Action potential upstroke velocity and maximum diastolic potential were also reduced. Under *I*_B,Na_‐selective conditions, 10 *μ*mol/L SKF‐96365 inhibited *I*_B,Na_ at −50 mV by 36.1 ± 6.8% (*n* = 8); however, effects on additional channel currents were also observed. Thus, the peak l‐type calcium current (*I*_Ca,L_) at +10 mV was inhibited by 38.6 ± 8.1% (*n* = 8), while the rapid delayed rectifier current, *I*_Kr_, tails at −40 mV following depolarization to +20 mV were inhibited by 55.6 ± 4.6% (*n* = 8). The hyperpolarization‐activated current, *I*_f_, was unaffected by SKF‐96365. Collectively, these results indicate that SKF‐96365 exerts a moderate inhibitory effect on *I*_B,Na_ and slows AVN cell pacemaking. However, additional effects of the compound on *I*_Ca,L_ and *I*_Kr_ confound the use of SKF‐96365 to dissect out selectively the physiological role of *I*_B,Na_ in the AVN.

## Introduction

The atrioventricular node (AVN) is a small but important component of the cardiac pacemaker conduction system; slow impulse conduction through the AVN coordinates the normal sequence of atrial and ventricular excitation and can protect the ventricles from too fast a rate during supraventricular tachycardias (Childers 1977; Meijler and Janse 1988). The AVN can also act as a subsidiary pacemaker, should the primary pacemaker, the sinoatrial node (SAN), fail (Childers 1977; Meijler and Janse 1988). The ionic basis of AVN cell pacemaking is incompletely understood, but is considered to involve multiple ionic conductances (Hancox et al. 2003). AVN cells lack significant inwardly rectifying K^+^ current at diastolic potentials and have a high membrane resistance, meaning that relatively small currents can have a significant effect on membrane potential (e.g., Noma et al. 1984; Hancox et al. 1993; Yuill and Hancox 2002; Choisy et al. 2015). The hyperpolarization‐activated current, *I*_f_, is present in a proportion of AVN cells from the rabbit, albeit at a lower density than in the primary pacemaker, the SAN (Nakayama et al. 1984; Hancox and Levi 1994b; Habuchi et al. 1995; Munk et al. 1996). Inhibition of *I*_f_ slows but does not arrest spontaneous activity in the intact AVN (Dobrzynski et al. 2003), suggestive of an important but not an obligatory role of this current. Inhibitors of intracellular calcium cycling and sodium‐calcium exchange (NCX) can arrest activity of isolated AVN cells and slow the activity of intact spontaneously active AVN preparations from multiple species (Nikmaram et al. 2008; Ridley et al. 2008; Kim et al. 2010; Cheng et al. 2011, 2012). Inhibitors of the rapid delayed rectifier current, I_Kr_, also alter spontaneous rate (Sato et al. 2000; Yamazaki et al. 1996) and the evidence from genetically modified mice additionally implicates Cav 1.3 and 3.1 calcium channels in AVN automaticity (Marger et al. 2011). Thus, multiple overlapping current components have been identified that contribute to AVN cell automaticity.

A notable feature of AVN cellular electrophysiology under experimental voltage clamp is that small tissue or single‐cell AVN preparations exhibit a “zero‐current” potential of ~−40 mV (e.g., Taniguchi et al. 1981; Hancox et al. 1993; Martynyuk et al. 1995; Hancox et al. 2003). As this membrane potential lies somewhat positive to the equilibrium potential for K^+^ ions, this observation suggests that AVN cells have an inward background current component. Consistent with this, through the study of AVN cells with major time‐ and voltage‐dependent conductances inhibited, an inward background sodium current (*I*_B,Na_) has been recently identified that is partially inhibited by lanthanides and low pH (Cheng et al. 2016). The current flows through nonselective cation channels (NSCCs), exhibiting a permeability sequence similar to that reported previously for an analogous current found in SAN cells (Hagiwara et al. 1992). Fluctuation analysis suggests that the channels underlying *I*_B,Na_ are of low conductance (3.2 pS; Cheng et al. 2016). Atrioventricular node cell action potential simulations have suggested that *I*_B,Na_ can influence significantly spontaneous action potential rate (Cheng et al. 2016), although a lack of selective pharmacology has precluded direct experimental validation of this. SKF‐96365 (1‐[*β*‐(3‐(4‐methoxyphenyl)propoxy)‐4‐methoxyphenethyl]‐1*H*‐imidazole hydrochloride) is a widely used inhibitor of NSCCs (Alexander et al. 2009). It has been reported to decrease the mouse SAN spontaneous rate (Ju et al. 2007) and a recent study of the developing chick heart reported that SKF‐96365 produced negative chronotropic and dromotropic (first and second degree atrioventricular block) effects (Sabourin et al. 2011). To our knowledge, however, no study has hitherto investigated directly the effects of this NSCC inhibitor on AVN cellular electrophysiology; this information is essential for the determination of the compound's utility for studying the physiological role of the *I*_B,Na_. This study was undertaken to address this deficit in information, with the results providing evidence that this agent affects spontaneous activity and inhibits AVN cation conductances, including but not restricted to *I*_B,Na_.

## Methods

### AVN cell isolation

AVN cells were isolated from the hearts of male New Zealand White rabbits (2–3 kg) killed humanely in accordance with UK Home Office Legislation. Cells were isolated from the entire AVN region from within the triangle of Koch, identified in relation to anatomical landmarks (Hancox et al. 1993; Cheng et al. 2009). The mechanical and enzymatic dispersion method used has been described previously (Hancox et al. 1993; Cheng et al. 2009). Isolated cells were stored in refrigerated Kraftbrühe (“KB”) solution (Isenberg and Klockner 1982; Hancox et al. 1993) until they were used.

### Electrophysiological recording

The experimental chamber for electrophysiological recording was mounted on the stage of an inverted microscope (Eclipse TE2000‐U, Nikon, Japan). Isolated cells were placed in this chamber and superfused with a standard Tyrode's solution containing (in mmol/L): 140 NaCl, 4 KCl, 2 CaCl_2_, 1 MgCl_2_, 10 glucose, 5 HEPES (pH 7.4 with NaOH). Patch pipettes were pulled and heat polished to resistances of 2–3 MΩ. For action potential (AP) recordings, pipettes were filled with a solution containing (in mmol/L): 110 KCl, 10 NaCl, 10 HEPES, 0.4 MgCl_2_, 5 glucose, 5 K_2_ATP, 0.5 GTP–Tris (pH 7.1 with KOH) (Choisy et al. 2012, 2015). The pipette solution for net ionic current recordings (from which *I*_Ca,L_, *I*_Kr_, and *I*_f_ were derived) was similar, except that it also included 5 mmol/L K_4_BAPTA (BAPTA (1,2‐bis(o‐aminophenoxy)ethane‐N,N,N′,N′‐tetraacetic acid), tetrapotassium salt) (Choisy et al. 2012, 2015).

For measurements of *I*_B,Na_, the same solutions were used to those in prior measurements of this current from SAN and AVN cells (Hagiwara et al. 1992; Cheng et al. 2016). Na^+^‐containing external solution contained (in mmol/L): 150 NaCl, 5 HEPES, 2 CsCl, 2 NiCl_2_, 1 BaCl_2_, 1 MgCl_2_, 0.01 strophanthidin (pH 7.4 with Tris base), while for Na^+^‐free (Tris‐substituted) solution, NaCl was replaced with equimolar Tris base (pH 7.4 with HCl). The pipette solution for background current recording contained (in mmol/L): 120 CsOH, 20 CsCl, 5 HEPES, 10 EGTA, 5 K_2_‐creatine phosphate, 5 Mg‐ATP, 2 MgCl_2_, 100 aspartic acid (pH of 7.4 with CsOH). For all experiments, once the whole‐cell configuration had been attained, superfusates were applied (35–37°C) using a home‐built rapid solution exchange device (Levi et al. 1996).

Recordings were made using an Axopatch‐1D amplifier (Axon Instruments, Sunnyvale CA). Protocols were generated and data recorded online with pClamp 10.0 software (Molecular Devices, Sunnyvale, CA) via an analog‐to‐digital converter Digidata 1322 (Molecular Devices). During AP recording, the AP digitization rate was 2 kHz. Membrane currents recorded in whole‐cell voltage‐clamp mode were digitized at 10 kHz with an appropriate bandwidth set on the recording amplifier. Data are presented as mean ± SEM. A statistical analysis of drug effects was performed using a paired *t*‐test and one‐ or two‐way ANOVA with Bonferroni *post‐test*, as appropriate.

### SKF‐96365

SKF‐96365 was obtained from Sigma‐Aldrich (Poole, Dorset, UK). It was dissolved in distilled water to produce a stock solution of 10 mmol/L. Aliquots of this stock solution were added to external superfusate to a final concentration of 10 *μ*mol/L. This concentration is similar to that used in prior investigation of cardiac NSCC (Zhang and Hancox 2003) and matches closely the half‐maximal inhibitory concentration for reported atrioventricular conduction effects on chick hearts (10.3 *μ*mol/L; Sabourin et al. 2011).

## Results

### Effects on spontaneous activity

**Figure 1:**
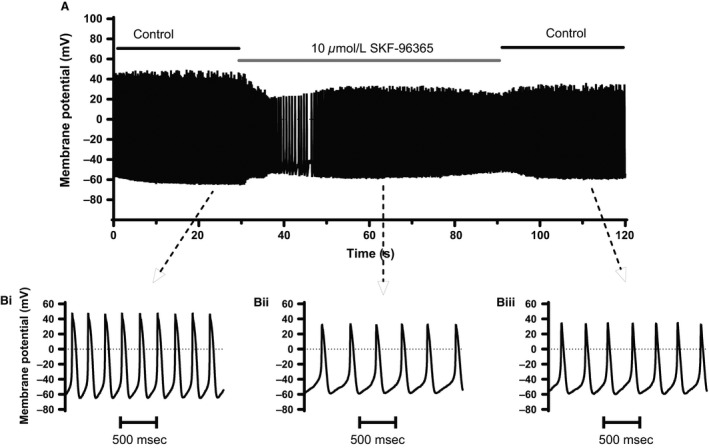
Effect of SKF‐96365 on spontaneous action potentials. (A) shows a representative slow time‐base recording of membrane potential from an AVN myocyte before, during, and after application of 10 *μ*mol/L SKF‐96365 as indicated above the trace. (B) displays at a faster time‐base sections of the top panel from the periods indicated, in the control solution (Bi), during application of SKF‐96365 (Bii), and following washout with control solution (Biii). Tabulated mean AP (action potential) parameters are included in Table 1. AVN, atrioventricular node.

Spontaneous APs were acquired continuously with the gap‐free acquisition mode by current clamping with a zero current input. Figure 1 shows representative results from a single experiment. The slow time‐base recording in panel A shows that application of SKF‐96365 rapidly led to a reduction in AP overshoot and a depolarization of maximum diastolic potential (MDP). Figures1Bi–Biii show portions of this record on an expanded time scale. Comparison of Figure1Bi (in control solution) with Figure1Bii (in SKF‐96365) shows that in addition to a decrease in AP amplitude, the spontaneous AP rate was also decreased. Figure1Biii shows APs following washout of SKF‐96365 in the same cell as Figures1Bi and Bii, illustrating partial recovery toward control. Table 1 summarizes mean AP parameters from a total of eight similar experiments in control and in SKF‐96365. Both spontaneous AP rate and the slope of the diastolic depolarization were significantly reduced in SKF‐96365 and, additionally, overshoot amplitude and MDP were also significantly reduced (leading to a significant reduction in AP amplitude). Furthermore, both the upstroke velocity and repolarization velocity of APs were significantly smaller in SKF‐96365, with a modest increase in AP duration at 50% repolarization (APD_50_). Thus, SKF‐96365 exerted multiple effects on spontaneous APs from AVN cells.

**Table 1:** Effect of SKF‐96365 on spontaneous action potential (AP) in rabbit atrioventricular node cells

Parameters	Control	10 *μ*mol/L SKF‐96365
Spontaneous AP rate (beats sec^−^^1^)	4.05 ± 0.39	3.50 ± 0.40*
(Percentage decrease compared with Control) Slope of pacemaker diastolic depolarization (mV sec^−^^1^)	158.1 ± 30.5	(13.9 ± 5.3%#) 86.8 ± 20.6 **
Maximal upstroke velocity (*V*_max_, *V* sec^−^^1^)	9.8 ± 2.3	5.6 ± 1.1**
Maximal repolarization velocity (*V*_rep_, *V* sec^−^^1^)	−1.8 ± 0.1	−1.4 ± 0.1**
AP duration at 50% repolarization (APD_50_, msec)	49.1 ± 2.5	53.3 ± 2.4 *
Maximal diastolic potential (MDP, mV)	−60.5 ± 2.7	−56.4 ± 2.9**
Overshoot (mV)	25.0 ± 4.1	13.7 ± 4.1**
AP amplitude (mV)	85.5 ± 4.8	70.2 ± 4.9**

The tabulated data were derived from eight experiments. Paired *t*‐test: *P<0.05, **P<0.01 versus Control. One sample *t*‐test: ^#^ *P*<0.05 compared with 0 change.

### Effect on *I*_B,Na_

**Figure 2:**
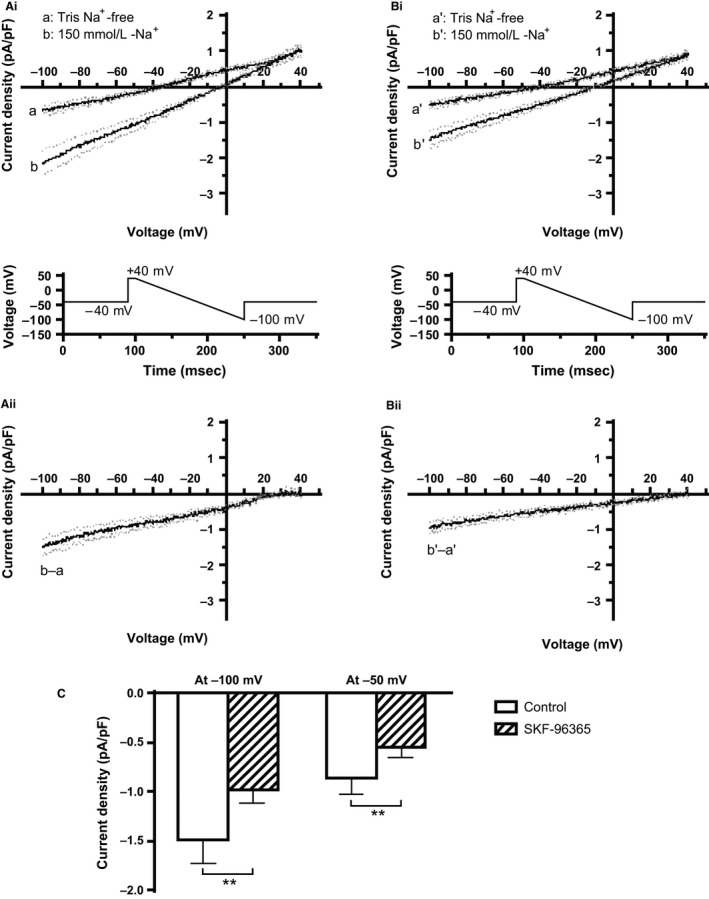
Effect of SKF‐96365 on the sodium‐dependent background current (*I*_B,Na_). (Ai, Bi): Mean currents in 150 mmol/L‐Na^+^ (b and b') and Tris Na^+^‐free (a and a') solutions (±SEM shown as gray dotted lines; *n* = 8 cells). The descending voltage ramp protocol used for these experiments is shown underneath each panel. (Ai) shows the control condition, and Bi is in the presence of 10 *μ*mol/L SKF‐96365. (Aii, Bii) I–V relations for Na^+^‐dependent I_B,Na_ (150 mmol/L‐Na^+^ minus Tris Na^+^‐free in Ai and Bi). (Aii) shows the control condition and (Bii) is in the presence of 10 *μ*mol/L SKF‐96365. (C) Bar charts show extent of inhibition of *I*_B,Na_ by 10 *μ*mol/L SKF‐96365. At −100 mV, *I*_B,Na_ was inhibited by 32.1 ± 5.3 %, and at −50 mV, *I*_B,Na_ was inhibited by 36.1 ± 6.8%. **P < 0.01, *n* = 8.

An inward background sodium current, *I*_B,Na_, was measured under the selective conditions described in the Methods (see also Cheng et al. 2016 and Hagiwara et al. 1992), as the difference in current between 150 mmol/L sodium and Tris‐containing extracellular superfusates. The protocol used to elicit *I*_B,Na_ was a descending voltage ramp between +40 and −100 mV (shown below Fig. 2Ai and Bi) at a frequency of 0.2 Hz. Figure2Ai shows mean (±SEM) currents in Na‐ and Tris‐containing solutions elicited by the voltage ramp protocol in the absence of SKF‐96365, while Figure2Bi shows comparable measurements in the presence of 10 *μ*mol/L SKF‐96365. For each cell studied, *I*_B,Na_ was obtained as the Na‐Tris difference current and the currents from different experiments were then normalized to cell capacitance and pooled for eight experiments. Figure2Aii shows the mean resulting *I*_B,Na_ in control conditions, while Figure2Bii shows *I*_B,Na_ obtained following treatment with SKF‐96365. The amplitude of *I*_B,Na_ was reduced in the presence of SKF‐96365. To determine whether this reduction was statistically significant, the amplitude of *I*_B,Na_ was compared between control and SKF‐96365 at two voltages (Fig. 2C): −100 mV (the most negative voltage in the examined range, at which *I*_B,Na_ amplitude was largest) and −50 mV (a potential within the diastolic depolarization range). At both voltages, the reduction in *I*_B,Na_ by SKF‐96365 was statistically significant (*P *< 0.01).

### Effects on *I*_Ca,L_, *I*_Kr_, and *I*_f_

**Figure 3:**
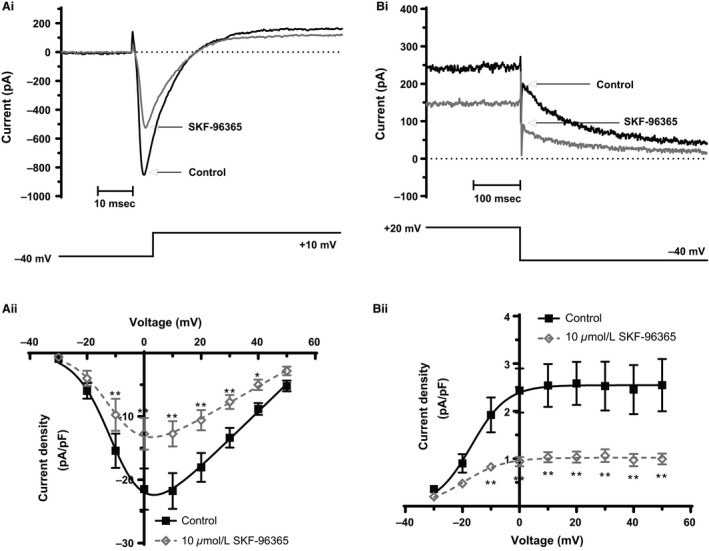
Effect of SKF‐96365 on *I*_Ca,L_ and *I*_Kr_. **(**Ai) Representative *I*_Ca,L_ records from an AVN cell before and during application of 10 *μ*mol/L SKF‐96365. The voltage protocol is shown below the current traces. (Aii) Current–voltage (I–V) relations for *I*_Ca,L_ (normalized to membrane capacitance). Mean (±SEM) data are shown for eight cells in control and with application of SKF‐96365. **(**Bi) Representative *I*_Kr_ records from an AVN cell before and during application of 10 *μ*mol/L SKF‐96365. The voltage protocol is shown below the current traces. (Bii) Current–voltage (I–V) relations for *I*_Kr_ (normalized to membrane capacitance). Mean (±SEM) data are shown for eight cells in control and in the presence of SKF‐96365 solutions. *P < 0.05, **P < 0.01 repeated‐measures two‐way ANOVA with Bonferroni post‐test (Aii and Bii). AVN, atrioventricular node.

Net ionic currents were recorded using K^+^‐based, BAPTA‐containing pipette solution and a protocol comprised of 500 msec voltage commands applied to a range of test potentials between −120 mV and +50 mV (at 0.2 Hz). This protocol and recording conditions have been used in prior AVN studies from our laboratory to study *I*_Ca,L_, *I*_Kr_, and *I*_f_ (Cheng et al. 2009; Choisy et al. 2012, 2015). The l‐type calcium current *I*_Ca,L_ was elicited by depolarizing commands from −40 mV to more positive voltages, with peak current occurring at 0/+10 mV. Figure3Ai shows representative *I*_Ca,L_ records in control superfusate and following application of 10 *μ*mol/L SKF‐96365. The peak current was reduced by SKF‐96365 exposure. Figure3Aii shows mean current–voltage (I–V) relations in control and SKF‐96365, which deviated from one another significantly between −10 and +40 mV. A fit to the data with a modified Boltzmann equation (Choisy et al. 2012, 2015) gave *V*_0.5_ and *k* values of −9.2 ± 1.8 mV and 6.0 ± 0.2 mV, respectively, for control and −9.3 ± 2.2 mV and 5.9 ± 0.4 mV with SKF‐96365 (*P *> 0.8 and 0.7, respectively; *n* = 8). The mean inhibition of peak *I*_Ca,L_ at +10 mV was 38.6 ± 8.1% (*n* = 8) and in the range of potentials over which the I–V relations in Figure3 Aii significantly diverged, there was no significant voltage dependence of fractional inhibition of *I*_Ca,L_ (ANOVA, *P *> 0.9; *n* = 8).

Rabbit AVN cells exhibit *I*_Kr_, but lack the slow delayed rectifier current, *I*_Ks_ (Habuchi et al. 1995; Howarth et al. 1996; Cheng et al. 2009): *I*_Kr_ tails on repolarization to −40 mV following depolarizing voltage commands are completely abolished by exposure to the selective *I*_Kr_ inhibitor E‐4031 (Howarth et al. 1996; Cheng et al. 2009). Consequently, the effects of SKF‐96365 on *I*_Kr_ were assessed by investigating the effects on outward tail currents following the depolarizing commands of the voltage protocol. Figure3Bi shows *I*_Kr_ tails on repolarization to −40 mV from +20 mV. The exposure to SKF‐96365 reduced the *I*_Kr_ tail amplitude markedly. Figure3Bii shows mean I–V relations for the *I*_Kr_ tail in control solution and SKF‐96365, with a significant suppression of the *I*_Kr_ amplitude between −10 and +50 mV. A fit to the data with a modified Boltzmann equation (Choisy et al. 2012, 2015) gave *V*_0.5_ and *k* values of −16.9 ± 1.5 mV and 5.7 ± 0.3 mV, respectively, for control and −19.4 ± 1.2 mV and 7.0 ± 1.2 mV with SKF‐96365 (*P *> 0.2 and 0.3, respectively; *n* = 8). *I*_Kr_ tails at −40 mV following depolarization to +20 mV were inhibited by 55.6 ± 4.6% (*n* = 8) and in the range of potentials over which the I–V relations in Figure3Bii significantly diverged, there was no significant voltage dependence of fractional inhibition of *I*_Kr_ (ANOVA, *P *> 0.3; *n* = 8).

**Figure 4:**
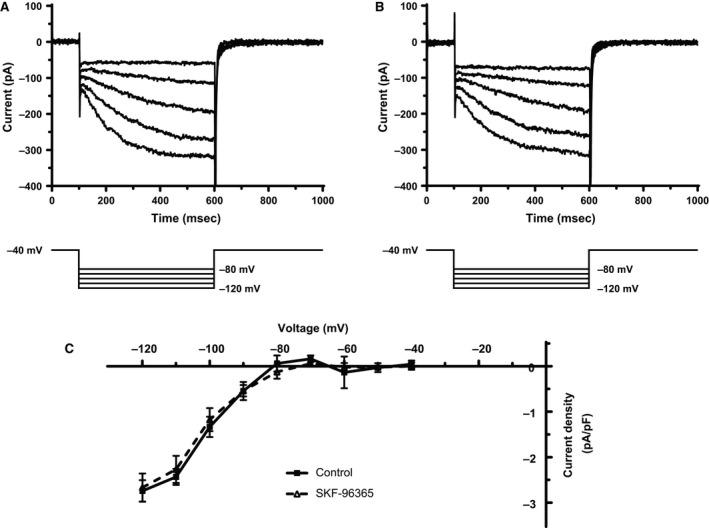
Effects of SKF‐96365 on hyperpolarisation activated current, *I*_*f*_. (**A,** B) In each panel, the upper traces show ionic currents activated by 500 msec duration hyperpolarizing voltage clamp commands applied from a holding potential of −40 mV (lower traces in each panel). Currents activated by commands to −80, −90, −100, −110, and −120 mV are shown. “A” shows records obtained in control condition, while “B” shows records from same cell obtained in the presence of 10 *μ*mol/L SKF‐96365. (**C)** I–V relationship for time‐dependent *I*_f_ (as end‐pulse minus start‐of‐pulse current) in control and with 10 *μ*mol/L SKF‐96365 (mean ± SEM, *n* = 5).

The hyperpolarization‐activated current, *I*_f_, can be elicited from rabbit AVN cells by hyperpolarizing voltage commands (Nakayama et al. 1984; Hancox and Levi 1994b; Habuchi et al. 1995; Munk et al. 1996); it can be quantified as the time‐dependent component of current at negative voltages, using the protocol employed in this study (Cheng et al. 2009; Choisy et al. 2012). Figure 4A and B show, respectively, representative currents elicited at voltages between −80 and −120 mV in control superfusate and with superfusate containing 10 *μ*mol/L SKF‐96365. The currents in the two conditions closely resembled one another. Figure 4C shows mean I–V relations for the time‐dependent (end pulse minus start pulse) *I*_f_ density during the protocol from a total of five experiments. At no voltage did this current differ between control and SKF‐96365. Thus, in contrast to *I*_Ca,L_ and *I*_Kr_, *I*_f_ was unaffected by SKF‐96365.

## Discussion

The principal motivation for this study was the lack of a small molecule inhibitor of cardiac *I*_B,Na_ that could be used to study the physiological role(s) of this current in cells from the AVN and, potentially, other cardiac regions. The inward background sodium current, *I*_B,Na_, is a comparatively understudied ionic conductance and the identification of a selective inhibitor would facilitate greatly the investigation of its physiological influence on activity from both the AVN and SAN. As *I*_B,Na_ is carried by NSCCs (Hagiwara et al. 1992; Cheng et al. 2016) and SKF‐96365 is a recognized NSCC inhibitor (Alexander et al. 2009), it was a plausible candidate to investigate for this purpose, particularly as it has been reported to influence AVN conduction (Sabourin et al. 2011). This study provides the first information on the actions of SKF‐96365 on AVN cellular electrophysiology, showing that the compound can alter spontaneous activity of AVN cells and that it can inhibit *I*_B,Na_. However, both our AP measurements and voltage‐clamp data indicate a lack of selectivity for *I*_B,Na_.

Prior efforts to characterize the influence of *I*_B,Na_ on the AVN have employed mathematical models of AVN cell and tissue electrophysiology (Cheng et al. 2016). A complete removal of *I*_B,Na_ from a spontaneously active cell model led to quiescence, while partial inhibition (by 60%) led to a slowing of AP rate accompanied by a modest hyperpolarisation of MDP, but without reduction in AP amplitude (Cheng et al. 2016). Additionally, the profile of stimulated APs in a one‐dimensional AVN tissue strand model was not affected by removal of *I*_B,Na_, but AP conduction velocity along the strand was slowed by 20% (Cheng et al. 2016). The results of these simulations were suggestive of roles for *I*_B,Na_ both in AVN cell pacemaker activity and in AVN conduction, without major effects on AP profile per se (Cheng et al. 2016). Against this background, the effects of SKF‐96365 on spontaneous APs in the present study are inconsistent with effects predicted for a selective action on *I*_B,Na_: significant effects of the compound were observed on AP amplitude, upstroke, duration, and depolarization of MDP (Fig. 1 and Table 1).

Under voltage clamp, 10 *μ*mol/L SKF‐96365 produced a partial inhibition of *I*_B,Na_ (by ~36% at −50 mV; Fig. 2). Higher concentrations were not tested against *I*_B,Na_ because this concentration also produced marked inhibition of both *I*_Ca,L_ and *I*_Kr_ (Fig. 3), indicating that the compound is at least as potent against the channels underlying these current as against those underlying *I*_B,Na_. The Cav1.3 l‐type channel isoform has been reported to predominate over Cav1.2 in the rabbit AVN (at the mRNA transcript level (Greener et al. 2009)). To our knowledge, there is no prior information on direct effects of SKF‐96365 on ionic currents carried by cardiac Cav1.2 or Cav1.3 channels. However, a prior study of frog skeletal muscle has reported partial inhibition of native l‐type channels with SKF‐96365 (Olivera and Pizarro 2010). Although recent data indicate that SKF‐96365 can also strongly inhibit ventricular sodium current, *I*_Na_, at low micromolar concentrations (Chen et al. 2015), Na channels are sparsely expressed in the central portion of the AVN (Petrecca et al. 1997). The L‐type calcium current, *I*_Ca,L_, is well established to contribute to AP genesis and conduction in the AVN (Zipes and Mendez 1973; Zipes and Fischer 1974; Hancox and Levi 1994a) and effects of SKF‐96365 on this current are therefore likely substantially to underlie the slowing of AP upstroke velocity and decreased overshoot seen here with the compound.

The rapid delayed rectifier current, *I*_Kr_, is active during both the repolarization and diastolic depolarization phases of the waveform of spontaneous AVN APs (Mitcheson and Hancox 1999) and inhibitors of *I*_Kr_ have been reported to slow spontaneous AVN rate (Sato et al. 2000; Yamazaki et al. 1996). A very recent independent study has reported that recombinant hERG channels (which underlie native *I*_Kr_) are inhibited by SKF‐96365, with a half‐maximal inhibitory concentration of 3.4 *μ*mol/L and modest voltage dependence of block (Liu et al. 2016). Experiments on native *I*_Kr_ were not conducted in that study (Liu et al. 2016), but further effects on recombinant KCNQ1+KCNE1 (*I*_Ks_) and Kir2.1 (*I*_K1_) channels (neither of which contribute to rabbit AVN spontaneous activity) were seen (Liu et al. 2016). Thus, the present results and those of Liu et al. (2016) are complementary to one another in demonstrating effects of SKF‐96365 on both recombinant and native *I*_Kr_ channels. Inhibition of *I*_Kr_ can account for the effects of SKF‐96365 on AVN AP repolarization velocity, AP duration, and MDP seen here.

In conclusion, this study demonstrates for the first time that SKF‐96365 partially inhibits the sodium‐dependent background current, *I*_B,Na_, in cells from the cardiac AVN. However, the compound also exerts marked effects on *I*_Ca,L_ and *I*_Kr_ and this precludes the use of SKF‐96365 for the selective investigation of *I*_B,Na_. Moreover, taken together with the results of other recent studies (Chen et al. 2015; Liu et al. 2016), the findings of the present investigation suggest that caution should be exercised in the use of SKF‐96365 to study the physiological contribution of cardiac NSCCs, as results obtained with the compound may, wholly, or in part, be attributable to off‐target actions on other cardiac channels.

## Conflicts of Interest

None.
